# Stevia and Stevioside Attenuate Liver Steatosis through PPARα-Mediated Lipophagy in *db/db* Mice Hepatocytes

**DOI:** 10.3390/antiox11122496

**Published:** 2022-12-19

**Authors:** Miey Park, Anshul Sharma, Hana Baek, Jin-Young Han, Junho Yu, Hae-Jeung Lee

**Affiliations:** 1 Department of Food and Nutrition, College of BioNano Technology, Gachon University, Seongnam 13120, Gyeonggi-do, Republic of Korea; 2 Institute for Aging and Clinical Nutrition Research, Gachon University, Seongnam 13120, Gyeonggi-do, Republic of Korea

**Keywords:** non-alcoholic fatty liver disease, stevia, stevioside, lipophagy, PPARα

## Abstract

Lipophagy, a type of autophagy that breaks down lipid droplets, is essential in the regulation of intracellular lipid accumulation and intracellular free fatty acid levels in numerous organisms and metabolic conditions. We investigated the effects of *Stevia rebaudiana Bertoni* (S), a low-calorie sweetener, and stevioside (SS) on hepatic steatosis and autophagy in hepatocytes, as well as in *db/db* mice. S and SS reduced the body and liver weight and levels of serum triglyceride, total cholesterol, and hepatic lipogenic proteins. In addition, S and SS increased the levels of fatty acid oxidase, peroxisome proliferator-activated receptor alpha (PPARα), and microtubule-associated protein light chain 3 B but decreased that of sequestosome 1 (p62) in the liver of *db/db* mice. Additionally, Beclin 1, lysosomal associated membrane protein 1, and phosphorylated adenosine monophosphate-activated protein kinase protein expression was augmented following S and SS treatment of *db/db* mice. Furthermore, the knockdown of PPARα blocked lipophagy in response to SS treatment in HepG2 cells. These outcomes indicate that PPARα-dependent lipophagy is involved in hepatic steatosis in the *db/db* mouse model and that SS, a PPARα agonist, represents a new therapeutic option for managing associated diseases.

## 1. Introduction

Non-alcoholic fatty liver disease (NAFLD) is the most common liver ailment caused by the buildup of excess fat in liver cells rather than by alcohol [1]. Hepatic steatosis, or fatty liver disease, develops when the liver’s weight is more than 5% fat [2]. Fatty liver disease can be divided into two types: when the liver has fat buildup and no damage, it is called NAFLD; when the liver has fat buildup, hepatocellular injury, inflammation, and different degrees of fibrosis, it is called non-alcoholic steatohepatitis (NASH) [3]. NAFLD is significantly linked to insulin resistance and obesity and is detected in more than 76% of type 2 diabetes (T2D) patients [4]. In addition, patients with T2D are at significant risk of developing NASH [3]. Recent findings suggest that autophagy may boost the lipid metabolism. Therefore, autophagy is considered to have therapeutic potential in NAFLD [5,6].

Autophagy mediates not only the redistribution of valuable nutrients during starvation but also the treatment of excess or damaged small organs and invading microorganisms [7]. When nutrients are sufficient, cells store their energy reserves as neutral lipids, cholesteryl esters, and triglycerides in the lipid droplets (LDs). Owing to the dynamic fusion of LDs with other intracellular organelles and erroneously folded proteins [8] or infectious particles [9], these apparently inactive LDs have often been reflected as separate organelles [10]. 

Autophagy breaks down LDs through a process called lipophagy [10,11]. Given that starvation triggers autophagy, which results in the production of nutrients through the lysosomal decomposition of unnecessary cytoplasmic content, autophagy has been postulated to have a role in disintegrating LDs to release free fatty acids for the starving cells and aid in lipid metabolism [12].

*Stevia rebaudiana Bertoni* (S) is a natural sweetener that is 300-times sweeter than sucrose and has various health-promoting biological effects [13,14]. These biological effects are elicited by the plant leaf extract, which contains secondary metabolites, such as polyphenols and steviol glycosides, including stevioside (SS), rebaudioside A, and rebaudioside C [15]. SS is one of the main compounds in stevia extract and constitutes between 4% and 20% of dried leaves [16]. It exhibits non-caloric, anti-inflammatory [17], anti-tumor [14], anti-diarrheal [18], and antihypertensive [19] effects and is effective in the treatment of hyperlipidemia [20]. Studies have shown that S and SS have many health benefits; however, their effects on hepatic metabolism and autophagy remain unclear. Therefore, the purpose of this study was to investigate how S and SS affect NAFLD in HepG2 cells and *db/db* mouse models.

## 2. Materials and Methods

### 2.1. Reagents

S leaf extract was obtained from Pharminogen (Pharminogen Inc., Gyeonggi-do, Korea). The concentrate was lyophilized to remove moisture completely and stored at −20 °C. SS (C_38_H_60_O_18_) was purchased from ChemFaces (ChemFaces Biochemical Co., Wuhan, China). Chloroquine (CQ) was procured from Sigma (Sigma-Aldrich Co., St. Louis, MO, USA), and the CYTO-ID Autophagy Detection Kit was acquired from Enzo Inc. (Enzo Life Sciences, Farmingdale, NY, USA).

### 2.2. HepG2 Cells Culture, Steatosis Induction, and Oil Red O Staining

HepG2 (human hepatoma) cells were acquired from the American Type Culture Collection (ATCC, Manassas, VA, USA). HepG2 cells were cultivated in Dulbecco’s modified Eagle’s medium (DMEM), which included 10% fetal bovine serum and 1% antibiotics (antibiotic-antimycotic, ThermoFisher, Waltham, MA, USA) at 37 °C in an environment with 5% CO_2_. Free fatty acid (FFA, oleic, and palmitic acid at a molar ratio of 2:1) stock was dissolved in isopropyl alcohol. 

To induce hepatic steatosis, HepG2 cells were grown in a serum-free medium and exposed to 1 mM FFA for 24 h. BSA (1%) was used as a control. HepG2 cells were fixed in formalin (10%) and stained with a working solution of Oil Red O for 30 min. After three washes, the cells were viewed under an inverted microscope, and images were taken. Following the observation, the absorbance of the lipids (in 1 mL of 100% isopropanol) was measured at 500 nm. Each experiment was conducted in triplicate.

### 2.3. Mouse Models and Diets

Male C57BL/6J *db/db* mice (BKS.Cg-Dock7m +/+ *Leprdb*/J, homozygote, 8-weeks-old) and negative controls (C57BL/6J mice, heterozygotes, similar age) were acquired from Jackson Laboratories (Sacramento, CA, USA). Following acclimatization for 2 weeks, the animals were randomly allocated into six groups as follows: the negative control group (C57BL/6J, N+; saline, *n* = 6), control group (C57BL/6J *db/db*, NC; saline, *n* = 6), positive control group (C57BL/6J *db/db*, PC, *n* = 6; saline with metformin 200 mg/kg/day), low S treatment group (C57BL/6J *db/db*, S200, *n* = 6; saline with 200 mg/kg/day of S extract), high S treatment group (C57BL/6J *db/db*, S500, *n* = 6; saline with 500 mg/kg/day of S extract), and SS treatment group (C57BL/6J *db/db*, SS, *n* = 6; saline with 40 mg/kg/day of SS). Mice were orally administered saline and SS (prepared in saline) during the three weeks. 

Three weeks later, all 36 mice were sacrificed. All studies using the chosen mouse models were approved by Gachon University (GI-ACUC-R2020012) and performed in compliance with the guidelines of the Guide for the Care and Use of Laboratory Animals.

### 2.4. Liver Tissue Histological Evaluation and Oil Red O Staining

Liver tissue samples from all the experimental mice groups were fixed in formalin (10%, Sigma-Aldrich, MO, USA) for a minimum of 72 h. The tissue sections (3–4 μm) were stained with hematoxylin-eosin (H-E) and Oil Red O solutions. A competent pathologist examined the stained sections using an Olympus Provis AX70 microscope (Olympus, Tokyo, Japan). A scale bar of 100 μm is used.

### 2.5. Real-Time PCR Quantification of Gene Expression

The total RNA was extracted from HepG2 cells and liver tissues using a commercial kit (iNtRON Biotechnology, Gyeonggi-do, Korea) in accordance with the owner’s manual. Following quantification, 0.8 μg of RNA was reverse transcribed into complementary DNA utilizing the iScript cDNA synthesis kit (Bio-Rad, Hercules, CA, USA). SYBR^®^ Green Master Mix (TaKaRa Bio, Otsu, Japan) was used in real-time PCR performed on a QuantStudio3 machine (Thermo Fisher Scientific). 

The following primers (5′–3′) were used: peroxisome proliferator-activated receptor gamma (PPARγ, NC_000072.7) forward CAGGAGAGCAGGGATTTGCA and reverse CCTACGCTCAGCCCTCTTCAT; sterol regulatory element-binding transcription factor-1c (SREBP-1c, NM_001113566.1) forward ATCGCAAACAAGCTGACCTG and reverse AGATCCAGGTTTGAGGTGGG; CCAAT/enhancer binding protein alpha (C/EBPα, NM_001287523.1) forward TTACAACAGGCCAGGTTTCC and reverse GGCTGGCGACATACAGTACA; fatty acid synthase (FAS, NC_000077.7) forward TTGCTGGCACTACAGAATGC and reverse AACAGCCTCAGAGCGACAAT; and β-actin (NM_007393.5) forward CTGTCCCTGTATGCCTCTG and reverse ATGTCACGCACGATTTCC. The expression levels of the chosen genes were compared to that of β-actin.

### 2.6. Western Blotting

For protein expression analysis, the total proteins were harvested from HepG2 cells and liver tissues using a lysis buffer for the extraction of proteins (iNtRON Biotechnology) comprising protease and phosphate inhibitors. Protein (30 μg) samples were electrophoretically separated on a polyacrylamide gel containing 15% sodium dodecyl sulfate and electrophoretically shifted to polyvinylidene difluoride membranes (Bio-Rad Laboratories, Hercules, CA, USA). 

After blocking with 5% skim milk at room temperature (RT, 18 to 25 °C), the membranes were incubated for more than 2 h with the following primary antibodies at RT: PPARγ (1:1000 dilution), SREBP-1c (1:1000 dilution), C/EBPα (1:500 dilution), FAS (1:1000 dilution), microtubule-associated protein light chain 3 B (LC3B; 1:1000 dilution), sequestosome 1 (SQSTM1; 1:1000 dilution), TFEB (1:1000 dilution), CPT-1 (1:1000 dilution), lysosomal associated membrane protein 1 (LAMP-1; 1:1000 dilution), BECN1 (1:1000 dilution), Bcl-2 (1:1000 dilution), Bax (1:1000 dilution), PPARα (1:1000 dilution), adenosine monophosphate-activated protein kinase (AMPK; 1:1000 dilution), and p-AMPK (1:1000 dilution). 

The primary antibodies were acquired from Abcam (Cambridge, UK) and Santa Cruz Biotechnology (Dallas, TX, USA). After 1 h of incubation at RT with the secondary antibody, the membranes were developed using the Miracle-Star™ Western Blot Detection System (iNtRON Biotechnology) and photographed using the ImageQuant^TM^ LAS500 system (GE Healthcare Life Sciences, Issaquah, WA, USA). Densitometry data after western blot analysis were obtained using the Amersham Imager 680 analysis software (GE Healthcare Life Sciences, USA) and used for analysis.

### 2.7. Detection of Autophagy with CYTO-ID^®^ Green

Autophagy was detected using the Cyto-ID^®^ Autophagy Detection Kit (Enzo Life Sciences) in accordance with the owner’s manual. Briefly, steatosis-induced cells were stained by incubating them with Cyto-ID^®^ Green stain solution for 30 min at 37 °C in the dark. The samples were rinsed once, stained with Hoechst 33342 for 1 min, washed three times with PBS, and mounted onto slides. Fluorescence images were obtained using a fluorescence inverted phase-contrast microscope (KI-2000F, Korea Lab Tech, Gyeonggi, Korea) and inspected using processing software (OptiView 3.7, Korea Lab Tech).

### 2.8. Small Interfering RNAs (siRNAs) and Transfection

PPARα (Catalog No. AM51331) and negative control (NC, Catalog No. AM4635) siRNAs were obtained from Invitrogen (Waltham, MA, USA). One day before transfection, HepG2 cells were seeded in six-well plates at a density of 2 × 10^5^ cells/well. The following day, Opti-MEM (Gibco-BRL, Thermo Fisher Scientific) was used as a culture medium substitute. Both the siRNAs were prepared in an Opti-MEM medium and mixed with Lipofectamine^®^ RNAiMAX (7 μL) reagent (Thermo Fisher Scientific) to a final volume of 200 μL. The produced mixture was applied to each well after incubating for 20 min at room temperature (25 °C). Fresh medium was given to each well after 4 h of incubation, and cells were then allowed to grow. After 20 h, the transfected cells were treated with stevioside and harvested after 24 h.

### 2.9. Statistical Analysis

The mean ± standard error (SE) is used to express all experimental results. Each experiment was performed three times, GraphPad Prism 9.02 (GraphPad Software, USA) was used for statistical analysis using one-way analysis of variance (ANOVA), and Tukey’s post-hoc testing was used to compare multiple independent groups. Statistical significance was set at *p* < 0.05.

## 3. Results

### 3.1. S and SS Attenuated Liver Steatosis in db/db Mice

**Figure 1:**
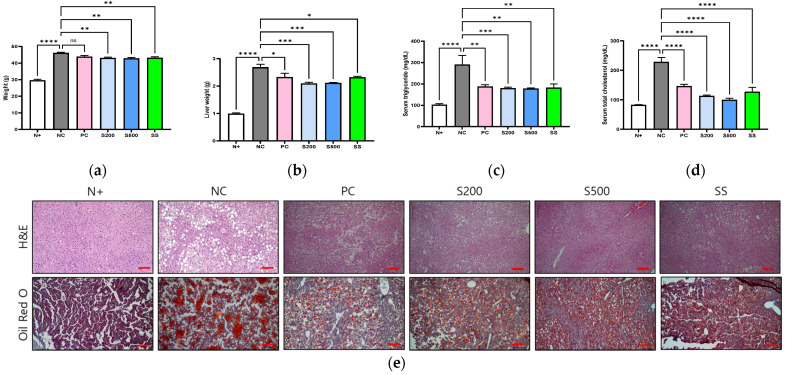
Stevia and stevioside attenuate lipid accumulation in *db/db* mice. Mice were orally administered saline (N+ and NC), saline with 200 mg/kg/day metformin (PC), saline with 200 or 500 mg/kg/day stevia (S200 and S500), and saline with 40 mM/kg/day stevioside (SS) for 3 weeks. (**a**) Body weight. (**b**) Liver weight. (**c**) Serum triglyceride (TG). (**d**) Serum total cholesterol (TC). (**e**) Image of liver tissues. All data are presented as the mean ± standard error (SE), *n* = 6 and represent results from three independent experiments. Scale bar, 100 μm. * *p* < 0.05, ** *p* < 0.01, *** *p* < 0.001, and **** *p* < 0.0001.

After three weeks of oral administration of S and SS, the terminal body weights of *db/db* mice in the NC group showed significantly increased weighLinet (*p* < 0.01) from that in the S (S200 and S500) and SS groups (Figure 1a). In addition, the liver tissue weights of *db/db* mice were significantly decreased in the S200 (*p* < 0.001), S500 (*p* < 0.001), and SS (*p* < 0.1) treatment groups relative to that in the NC group (Figure 1b). The *db/db* mice had the highest plasma serum triglyceride (TG) and total serum cholesterol (TC) levels among type 2 diabetic mouse models [21]. 

Considering these characteristics, in this study, the weight and serum TG and TC levels in the NC group were higher than in the N+ group of normal mice, and they were significantly reduced by the oral administration of S and SS (Figure 1c,d). Histological analyses of the livers from the six *db/db* mouse groups revealed that, compared to the N+ group, the NC group showed hepatocellular damage (H&E staining) and widespread LDs (Oil Red O staining), whereas the S200, S500, and SS treatment groups showed decreased accumulation of LDs (Figure 1e).

### 3.2. S and SS Attenuated Expressions of Hepatic Lipid Genes in db/db Mice

**Figure 2:**
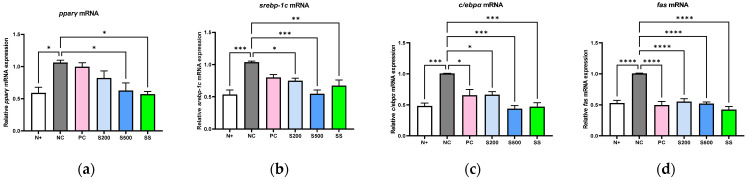

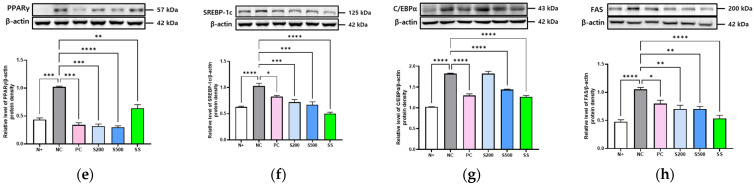
Stevia and stevioside attenuate adipogenic-related genes and proteins in *db/db* mice. Mice were orally administered saline (N+ and NC), saline with 200 mg/kg/day metformin (PC), saline with 200 or 500 mg/kg/day stevia (S200 and S500), and saline with 40 mM/kg/day stevioside (SS) for 3 weeks. (**a**,**e**) Peroxisome proliferator-activated receptor gamma (PPARγ). (**b**,**f**) Sterol regulatory element-binding transcription factor-1c (SREBP-1c). (**c**,**g**) CCAAT/enhancer binding protein alpha (C/EBPα). (**d**,**h**) Fatty acid synthase (FAS). All data are presented as the mean ± SE, *n* = 6 and represent results from three independent experiments. * *p* < 0.05, ** *p* < 0.01, *** *p* < 0.001, and **** *p* < 0.0001.

To examine the biological mechanisms of S in the liver, we used quantitative PCR and immunoblot analysis to examine the lipogenic markers. As shown in Figure 2, the mRNA expressions of PPARγ (*p* < 0.1), SREBP-1c (*p* < 0.001), C/EBPα (*p* < 0.001), and FAS (*p* < 0.0001) were significantly higher in the NC group relative to the N+ group (Figure 2a–d). The corresponding mRNA expression levels in the S200, S500, and SS groups were significantly lower than those in the NC group. Consistent with this, protein expression analysis also showed a significant decrease in the levels of the respective proteins in the S200-, S500-, and SS-treated groups (Figure 2e–f).

### 3.3. S and SS Activated Lipid Metabolism and AMPK Phosphorylation in db/db Mice

**Figure 3:**
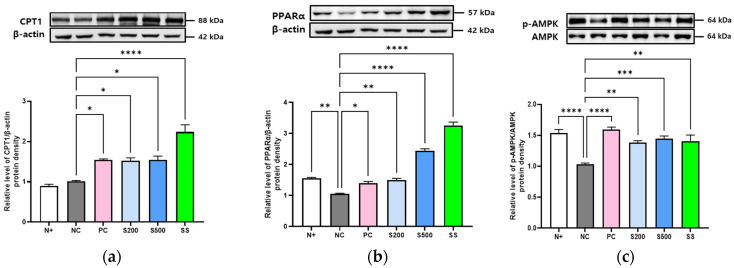
Stevia and stevioside activate fatty acid oxidation-related proteins in *db/db* mice. Mice were orally administered saline (N+ and NC), saline with 200 mg/kg/day metformin (PC), saline with 200 or 500 mg/kg/day stevia (S200 and S500), and saline with 40 mM/kg/day stevioside (SS) for 3 weeks. (**a**) Carnitine palmitoyl transferase-1 (CPT-1). (**b**) Peroxisome proliferator-activated receptor alpha (PPARα). (**c**) Adenosine monophosphate-activated protein kinase (AMPK). All data are presented as the mean ± SE, *n* = 3 and represent results from three independent experiments. * *p* < 0.05, ** *p* < 0.01, *** *p* < 0.001, and **** *p* < 0.0001.

The expression genes linked to fatty acid oxidation, such as PPARα and CPT1, were evaluated in the livers of *db/db* mice following S and SS treatment. Immunoblot analysis revealed enhanced levels of CPT1 and PPARα protein in response to S and SS treatment compared to those in the NC group (Figure 3a,b). In addition, the NC group showed a significant reduction in AMPK phosphorylation, which showed an increasing trend following S and SS treatment and was the highest in the PC group administered metformin (Figure 3c).

### 3.4. S- and SS-Induced Autophagy in Liver of db/db Mice

**Figure 4:**
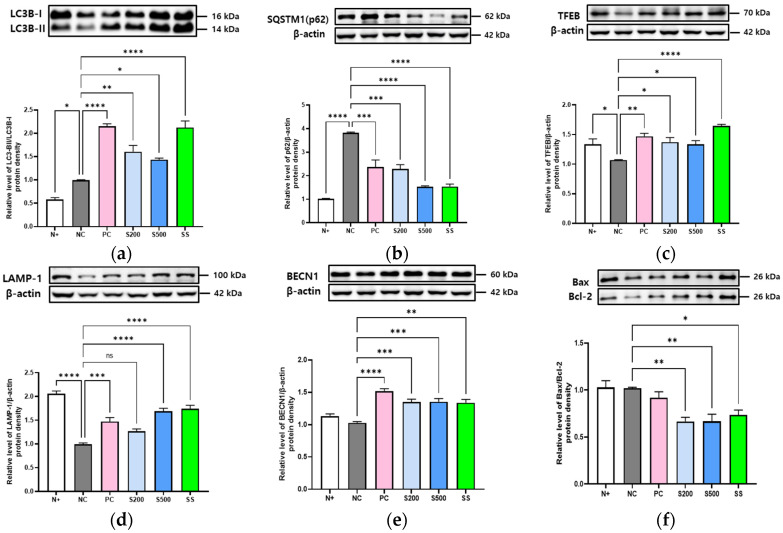
Stevia and stevioside induce autophagy in hepatocytes of *db/db* mice. Mice were orally administered saline (N+ and NC), saline with 200 mg/kg/day metformin (PC), saline with 200 or 500 mg/kg/day stevia (S200 and S500), and saline with 40 mM/kg/day stevioside (SS) for 3 weeks. (**a**) Microtubule-associated protein light chain 3B (LC3B)-II/LC3B-I. (**b**) Sequestosome1 (SQSTM1). (**c**) Transcription factor EB (TFEB). (**d**) Lysosomal associated membrane protein 1 (LAMP-1). (**e**) Beclin 1 (BECN1). (**f**) Bax/Bcl-2. All data are presented as the mean ± SE, *n* = 3 and represent results from three independent experiments. * *p* < 0.05, ** *p* < 0.01, *** *p* < 0.001, and **** *p* < 0.0001.

Next, we analyzed whether S and SS regulate autophagy to mediate intracellular lipid storage in the *db/db* mice liver tissue. We found that the expression of microtubule-associated protein light chain 3 B (LC3B)-II/LC3B-I was significantly increased in the S- (S200 (*p* < 0.01) and S500 (*p* < 0.1)) and SS-treated (*p* < 0.0001) groups compared to that in the NC group, whereas SQSTM1 (p62 and sequestosome1) was significantly downregulated in the S- (S200 (*p* < 0.001) and S500 (*p* < 0.0001)) and SS-treated (*p* < 0.0001) groups (Figure 4a,b). 

Analysis of the lysosome-related proteins, transcription factor EB (TFEB), and lysosome-associated membrane protein 1 (LAMP-1) revealed that TFEB expression was lower in the livers of *db/db* mice compared with in the normal (N+) group, although oral S and SS administration for three weeks markedly enhanced the relative levels of TFEB/β-actin protein (Figure 4c). Similar results were observed for LAMP-1 expression by immunoblot analysis (Figure 4d). The expression of Beclin 1 (BECN1) protein, which promotes crosstalk between apoptosis and autophagy, was increased in the livers of the S200, S500, and SS groups relative to that in the *db/db* mice (NC) group (Figure 4e). Furthermore, the relative Bax/Bcl-2 protein levels were lower in the S200, S500, and SS groups than those in the NC group (Figure 4f).

### 3.5. SS-Induced Autophagy in Steatosis-Induced Hepatocytes

**Figure 5:**
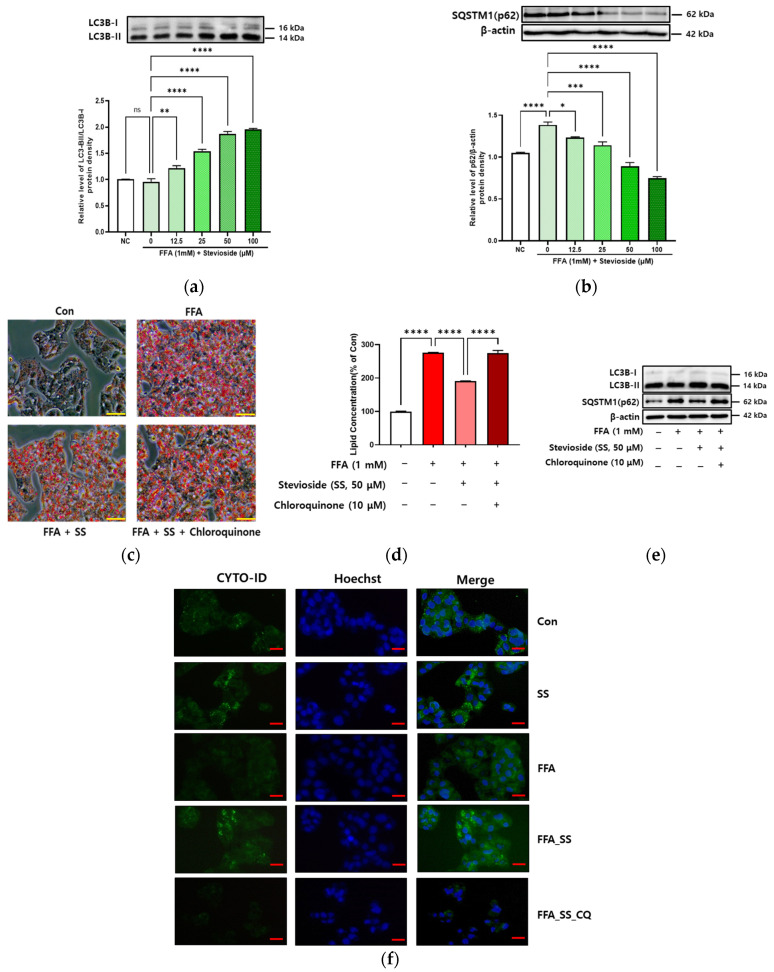
Stevioside induces autophagy in steatosis-induced hepatocytes. HepG2 cells were treated with 1 mM FFA and different concentrations of stevioside (SS; 0, 12.5, 25, 50, or 100 μM) in the presence or absence of 10 μM chloroquine (CQ) for 24 h. (**a**) Microtubule-associated protein light chain 3B (LC3B)-II/LC3B-I. (**b**) Sequestosome1 (SQSTM1). (**c**) HepG2 cells were stained with Oil Red O. Scale bar, 100 μm. (**d**) Intracellular lipid accumulation (the absorbance of lipids was measured at 500 nm). (**e**) Immunoblot analysis. (**f**) Cells were observed by fluorescence microscopy. Scale bar, 10 μm. All data are presented as the mean ± SE and represent results from three independent experiments. * *p* < 0.05, ** *p* < 0.01, *** *p* < 0.001, and **** *p* < 0.0001.

Different concentrations of SS (0, 12.5, 25, 50, and 100 μM) and 1 mM FFA were applied to HepG2 cells for 24 h. SS treatment augmented LC3B-II/LC3B-I levels and reduced SQSTM1 (p62) levels in a dose-dependent manner (Figure 5a,b).

SS-treated HepG2 cells were treated with chloroquine (CQ), an autophagy inhibitor that prevents autophagosomes from attaching to lysosomes, and Oil Red O staining and immunoassays were used to assess increasing lipophagy (Figure 5c–e). HepG2 cells treated with FFA showed increased lipid concentration compared to the control (Con).

After FFA and SS (50 μM) treatment, the lipid concentration was decreased compared to that in the Con (Figure 5d). The lipid concentration was increased following FFA treatment after CQ treatment showed the same results with decreased LC3B-II/LC3B-I levels and increased SQSTM1 (p62) levels (Figure 5e). Next, the effect of SS on autophagy was evaluated in steatosis-induced HepG2 cells using fluorescence microscopy. The presence of autophagic green vacuoles was assessed using an autophagy detection kit in HepG2 cells treated with SS, or FFA and SS (Figure 5f). The cells were also stained with the nuclear stain Hoechst 33342. As shown in Figure 5f, SS treatment markedly increased the number of green autophagic vacuoles throughout the cells.

### 3.6. SS-Induced Autophagy Is Dependent on PPARα in Hepatocytes

**Figure 6:**
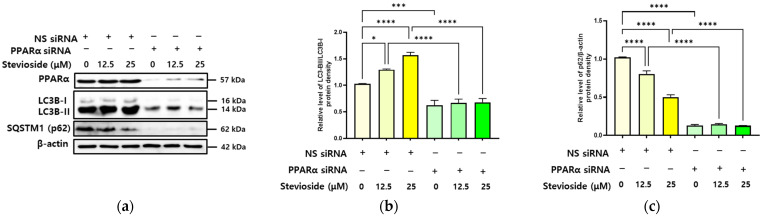
Stevioside-induced autophagy is dependent on PPARα in hepatocytes. HepG2 cells were transfected with the nonspecific (NS) siRNA or peroxisome proliferator-activated receptor α (PPARα) siRNA. After 24 h, the cells were treated with different concentration of stevioside (SS; 0, 12.5, and 25 μM) for another 24 h. (**a**) Immunoblot analysis of PPARα, microtubule-associated protein light chain 3B (LC3B)-II/LC3B-I, sequestosome1 (SQSTM1), and β-actin. (**b**) Relative expression of LC3B-II/LC3B-I. (**c**) Relative expression of SQSTM1(p62)/β-actin. All data are presented as the mean ± SE and represent results from three independent experiments. * *p* < 0.05, *** *p* < 0.001, and **** *p* < 0.0001.

To test our hypothesis that PPAR regulates lipophagy in hepatocytes, we used siRNAs to silence the PPAR gene in HepG2 cells. HepG2 cells transfected with the nonspecific (NS) siRNA showed significant autophagy induction following SS treatment, whereas cells with PPARα knockdown did not show any significant change in autophagy induction following SS treatment (Figure 6).

## 4. Discussion

In this study, we provide in vivo and in vitro evidence that S and SS induce autophagy and that the lysosomal pathway reduces liver steatosis and hepatic lipid gene expression in *db/db* mice and HepG2 cells. Furthermore, oral S and SS treatment reduces LDs and triggers fatty acid oxidation in the livers of *db/db* mice, which is controlled by autophagy and the lysosomal pathway. We also verified that PPAR plays a critical role in lipophagy, which reduces hepatocyte steatosis.

Appropriate amounts of lipids are essential for cellular functions and cell survival [12]. The finding that macrophages can break down some of the lipids in liver cells has opened up the possibility of controlling the pathologies associated with the lipid metabolism [22]. Lipid metabolism in the liver is associated with the plasma lipid levels, lipid synthesis, and lipid exports from the liver [23]. Increased LD levels in hepatocytes do not always cause cellular dysfunction [24]. 

Lipid metabolism regulates various cellular processes to produce energy or structural components in the cell membrane that regulate multiple cellular processes [25]. After oxidation to FFAs, LDs are destroyed via the mitochondrial fatty acid oxidation pathway to produce ATP (energy house) and meet the energy demands of rapidly growing cells [12,26]. 

In mice lacking leptin receptors (*db/db*), the levels of lipogenesis markers and LDs were significantly higher following a high-calorie intake for three weeks than those in normal control mice (Figure 1 and Figure 2). However, lipophagy and liver-specific AMPK activation were markedly lower than that in the other groups (Figure 3 and Figure 4), which differed significantly from the results of oral administration of S or SS in this study.

The transcriptional mechanism that links autophagy and the lipid metabolism is associated with the activation of nuclear TFEB [27]. Excessive expression of TFEB promotes the decomposition of autophagy substrates and immobilizes LDs and damaged mitochondria [28]. In an obese mouse model, TFEB-knockout resulted in lipid metabolism disorders and metabolic pathway imbalances [29]. TFEB controls genes involved in lipid metabolism through peroxisome proliferator-activated receptor gamma coactivator-1 alpha (PGC-1α) and PPARα, indicating that PGC-1α regulates lipid metabolism in the liver by controlling the activity of PPARα during starvation [29].

Fatty acids and their derivatives activate transcription factors, such as PPARs, helping to regulate the expression of genes [30]. Three subtypes of PPARs (PPARβ/δ, PPARα, and PPARγ) have been identified in mammals based on their tissue-specific expression patterns. PPARα is the most prevalent subtype in hepatocytes and is implicated in various lipid metabolic pathways [31]. PPARα is profusely expressed in tissues with high capabilities for fatty acid oxidation, such as the liver, heart, and skeletal muscles. This is an essential factor in converting and utilizing energy, particularly during fasting, and serves as the primary regulator of fatty acid homeostasis [32].

SS is the principal sweetening component of the *Stevia rebaudiana* leaf and constitutes 4–20% of the leaf (dry weight basis), depending on the cultivar and growing conditions [33]. In both animals and humans, SS is absorbed and metabolized without being degraded by digestive enzymes. SS is metabolized in the colon by the intestinal flora and absorbed into the portal vein by the colon wall as S, which is then partially transported to the liver, filtered by the kidneys, and eliminated in the urine as steviol glucuronide [34]. As the human intestinal enzymes cannot cleave the steviol structure, they are released without retention in the body. Therefore, steviol glycoside metabolites rarely accumulate in humans.

Several epidemiological studies have shown that consuming plant-based foods is beneficial in alleviating fatty liver diseases, including NAFLD [35,36,37]. Some fruits and their bioactive compounds ameliorate fatty liver disease by promoting the inhibition of apoptosis, inflammation, and oxidative stress and alleviate hepatic steatosis by regulating AMPK and SIRT1 signaling [38,39,40,41]. Specific plant-based foods with bioactive compounds are natural sources that prevent and alleviate fatty liver disease [42]. In addition, herbal bioactive compounds and medicinal plants complement a healthy lifestyle and appear to have several advantages in improving oxidative stress, cell inflammation, and insulin resistance in NAFLD treatment [43]. 

In this study, SS, a natural food compound, caused PPARα-mediated autophagy, which relieved fatty acid buildup in the liver, increased β-oxidation, and alleviated hepatic steatosis through the activation of lipophagy. Though many studies have found a connection between lipophagy and NAFLD, there is still some disagreement over the precise function of (members of the autophagy/lipophagy pathways) in NAFLD [44,45]. Pharmacological treatment has been shown to activate PPARα, which reverses the normal regulation of autophagy in the fed state and triggers lipophagy or autophagic lipid destruction [46]. Therefore, our goal was to determine whether or not PPAR was involved in the process of S and SS, causing an autophagic lipid degradation state (lipophagy).

According to studies, higher TFEB expression can boost lipophagy, which, in turn, can increase hepatic lipid catabolism and the beta-oxidation of fatty acids. Lysosomal biogenesis and autophagy are both regulated by TFEB [31]. A previous human investigation discovered that human liver tissues with both simple steatosis and steatohepatitis had lower TFEB expression than did the healthy controls [47]. In contrast to the control, the expression of this master regulator was elevated in our study after S and SS treatments. 

As can be observed in graphic abstract, increased expression of LC3-1 proteins can identify big lipid droplets (LD), attract them (often referred to as cargo), and help them fuse with the autophagolysosome membrane. Although p62 connects the cargo with the autophagosome, which makes it necessary for autophagy and lipophagy, increased levels typically signify accumulation or aggregation due to reduced autophagy [48]. A considerably lower expression of p62 was observed in our study after the treatment of S and SS. In the presence of LAMP-1, the newly formed autophagolysosome unites with the lysosome, playing a crucial part in this fusion process that creates the autolysosome. 

The liver content of LDs, TG, and TC in *db/db* mice treated with S and SS was lowered because the lysosomal acid lipase in the autolysosome converted TG and TC into free fatty acids. Free fatty acid then underwent beta-oxidation as indicated by enhanced PPARα and CPT-1 and decreased expression of SREBP-1c, FAS, PPARγ, and C/EBPα. Furthermore, the knockdown of PPARα blocked lipophagy in response to SS treatment in HepG2 cells. Beclin 1 function has been reported to be a cross regulator between autophagy and apoptosis. According to Kang and coworkers, Beclin 1 often interacts with a number of cofactors and allows for the creation of specific complexes that cause autophagy [49].

## 5. Conclusions

In the current study, we showed novel beneficial effects of S and SS, which ameliorate hepatic steatosis through lipophagy activation in the liver of *db/db* mice. Furthermore, the inhibition of PPARα, which plays an imperative role in fatty acid homeostasis in the liver, blocked the effects of SS. Our findings suggest that SS, a new lipophagy enhancer, represents a viable therapeutic option for hepatic steatosis.

## Data Availability

All of the data is contained within the article.

## References

[1] Abd El-Kader S.M., El-Den Ashmawy E.M.S. (2015). Non-alcoholic fatty liver disease: The diagnosis and management. World J. Hepatol..

[2] Nassir F., Rector R.S., Hammoud G.M., Ibdah J.A. (2015). Pathogenesis and Prevention of Hepatic Steatosis. Gastroenterol. Hepatol..

[3] Benedict M., Zhang X. (2017). Non-alcoholic fatty liver disease: An expanded review. World J. Hepatol..

[4] Portillo-Sanchez P., Bril F., Maximos M., Lomonaco R., Biernacki D., Orsak B., Subbarayan S., Webb A., Hecht J., Cusi K. (2015). High Prevalence of Nonalcoholic Fatty Liver Disease in Patients With Type 2 Diabetes Mellitus and Normal Plasma Aminotransferase Levels. J. Clin. Endocrinol. Metab..

[5] Mao Y., Yu F., Wang J., Guo C., Fan X. (2016). Autophagy: A new target for nonalcoholic fatty liver disease therapy. Hepatic Med. Evid. Res..

[6] Sinha R.A., Farah B.L., Singh B.K., Siddique M.M., Li Y., Wu Y., Ilkayeva O.R., Gooding J., Ching J., Zhou J. (2014). Caffeine stimulates hepatic lipid metabolism by the autophagy-lysosomal pathway in mice. Hepatology.

[7] Rogov V., Dötsch V., Johansen T., Kirkin V. (2014). Interactions between autophagy receptors and ubiquitin-like proteins form the molecular basis for selective autophagy. Mol. Cell.

[8] Cole N.B., Murphy D.D., Grider T., Rueter S., Brasaemle D., Nussbaum R.L. (2002). Lipid droplet binding and oligomerization properties of the Parkinson's disease protein alpha-synuclein. J. Biol. Chem..

[9] Filipe A., McLauchlan J. (2015). Hepatitis C virus and lipid droplets: Finding a niche. Trends Mol. Med..

[10] Martinez-Lopez N., Singh R. (2015). Autophagy and Lipid Droplets in the Liver. Annu. Rev. Nutr..

[11] Kounakis K., Chaniotakis M., Markaki M., Tavernarakis N. (2019). Emerging Roles of Lipophagy in Health and Disease. Front. Cell Dev. Biol..

[12] Zhang S., Peng X., Yang S., Li X., Huang M., Wei S., Liu J., He G., Zheng H., Yang L. (2022). The regulation, function, and role of lipophagy, a form of selective autophagy, in metabolic disorders. Cell Death Dis..

[13] Salehi B., López M.D., Martínez-López S., Victoriano M., Sharifi-Rad J., Martorell M., Rodrigues C.F., Martins N. (2019). *Stevia rebaudiana* Bertoni bioactive effects: From in vivo to clinical trials towards future therapeutic approaches. Phytother. Res. PTR.

[14] Iatridis N., Kougioumtzi A., Vlataki K., Papadaki S., Magklara A. (2022). Anti-Cancer Properties of *Stevia rebaudiana*; More than a Sweetener. Molecules.

[15] Purkayastha S., Markosyan A., Prakash I., Bhusari S., Pugh G., Lynch B., Roberts A. (2016). Steviol glycosides in purified stevia leaf extract sharing the same metabolic fate. Regul. Toxicol. Pharmacol. RTP.

[16] González C., Tapia M., Pérez E., Pallet D., Dornier M. (2014). Main properties of steviol glycosides and their potential in the food industry: A review. Fruits.

[17] Alavala S., Nalban N., Sangaraju R., Kuncha M., Jerald M.K., Kilari E.K., Sistla R. (2020). Anti-inflammatory effect of stevioside abates Freund’s complete adjuvant (FCA)-induced adjuvant arthritis in rats. Inflammopharmacology.

[18] Pariwat P., Homvisasevongsa S., Muanprasat C., Chatsudthipong V. (2008). A natural plant-derived dihydroisosteviol prevents cholera toxin-induced intestinal fluid secretion. J. Pharmacol. Exp. Ther..

[19] Ferri L.A., Alves-Do-Prado W., Yamada S.S., Gazola S., Batista M.R., Bazotte R.B. (2006). Investigation of the antihypertensive effect of oral crude stevioside in patients with mild essential hypertension. Phytother. Res. PTR.

[20] Ahmad U., Ahmad R.S., Arshad M.S., Mushtaq Z., Hussain S.M., Hameed A. (2018). Antihyperlipidemic efficacy of aqueous extract of *Stevia rebaudiana* Bertoni in albino rats. Lipids Health Dis..

[21] Nishina P.M., Lowe S., Wang J., Paigen B. (1994). Characterization of plasma lipids in genetically obese mice: The mutants obese, diabetes, fat, tubby, and lethal yellow. Metabolism.

[22] Parzych K.R., Klionsky D.J. (2014). An overview of autophagy: Morphology, mechanism, and regulation. Antioxid. Redox Signal..

[23] Nguyen P., Leray V., Diez M., Serisier S., Bloc’h J.L., Siliart B., Dumon H. (2008). Liver lipid metabolism. J. Anim. Physiol. Anim. Nutr..

[24] Mashek D.G. (2021). Hepatic lipid droplets: A balancing act between energy storage and metabolic dysfunction in NAFLD. Mol. Metab..

[25] Xie Y., Li J., Kang R., Tang D. (2020). Interplay Between Lipid Metabolism and Autophagy. Front. Cell Dev. Biol..

[26] Onal G., Kutlu O., Gozuacik D., Dokmeci Emre S. (2017). Lipid Droplets in Health and Disease. Lipids Health Dis..

[27] Li X., Zhang X., Zheng L., Kou J., Zhong Z., Jiang Y., Wang W., Dong Z., Liu Z., Han X. (2016). Hypericin-mediated sonodynamic therapy induces autophagy and decreases lipids in THP-1 macrophage by promoting ROS-dependent nuclear translocation of TFEB. Cell Death Dis..

[28] Moruno-Manchon J.F., Uzor N.E., Kesler S.R., Wefel J.S., Townley D.M., Nagaraja A.S., Pradeep S., Mangala L.S., Sood A.K., Tsvetkov A.S. (2016). TFEB ameliorates the impairment of the autophagy-lysosome pathway in neurons induced by doxorubicin. Aging.

[29] Settembre C., De Cegli R., Mansueto G., Saha P.K., Vetrini F., Visvikis O., Huynh T., Carissimo A., Palmer D., Klisch T.J. (2013). TFEB controls cellular lipid metabolism through a starvation-induced autoregulatory loop. Nat. Cell Biol..

[30] Montagner A., Polizzi A., Fouché E., Ducheix S., Lippi Y., Lasserre F., Barquissau V., Régnier M., Lukowicz C., Benhamed F. (2016). Liver PPARα is crucial for whole-body fatty acid homeostasis and is protective against NAFLD. Gut.

[31] Sinha R.A., Rajak S., Singh B.K., Yen P.M. (2020). Hepatic Lipid Catabolism via PPARα-Lysosomal Crosstalk. Int. J. Mol. Sci..

[32] Feige J.N., Gelman L., Michalik L., Desvergne B., Wahli W. (2006). From molecular action to physiological outputs: Peroxisome proliferator-activated receptors are nuclear receptors at the crossroads of key cellular functions. Prog. Lipid Res..

[33] Geuns J.M. (2003). Stevioside. Phytochemistry.

[34] Li Y., Zhu W., Cai J., Liu W., Akihisa T., Li W., Kikuchi T., Xu J., Feng F., Zhang J. (2021). The role of metabolites of steviol glycosides and their glucosylated derivatives against diabetes-related metabolic disorders. Food Funct..

[35] Xia H.M., Wang J., Xie X.J., Xu L.J., Tang S.Q. (2019). Green tea polyphenols attenuate hepatic steatosis, and reduce insulin resistance and inflammation in high-fat diet-induced rats. Int. J. Mol. Med..

[36] Yang Z., Zhu M.-Z., Zhang Y.-B., Wen B.-B., An H.-M., Ou X.-C., Xiong Y.-F., Lin H.-Y., Liu Z.-H., Huang J.-A. (2019). Coadministration of epigallocatechin-3-gallate (EGCG) and caffeine in low dose ameliorates obesity and nonalcoholic fatty liver disease in obese rats. Phytother. Res..

[37] Khoo W.Y., Chrisfield B.J., Sae-tan S., Lambert J.D. (2020). Mitigation of nonalcoholic fatty liver disease in high-fat-fed mice by the combination of decaffeinated green tea extract and voluntary exercise. J. Nutr. Biochem..

[38] Park M., Yoo J.H., Lee Y.S., Lee H.J. (2019). Lonicera caerulea Extract Attenuates Non-Alcoholic Fatty Liver Disease in Free Fatty Acid-Induced HepG2 Hepatocytes and in High Fat Diet-Fed Mice. Nutrients.

[39] Park M., Yoo J.H., Lee Y.S., Park E.J., Lee H.J. (2020). Ameliorative effects of black ginseng on nonalcoholic fatty liver disease in free fatty acid-induced HepG2 cells and high-fat/high-fructose diet-fed mice. J. Ginseng. Res..

[40] Liu Q., Pan R., Ding L., Zhang F., Hu L., Ding B., Zhu L., Xia Y., Dou X. (2017). Rutin exhibits hepatoprotective effects in a mouse model of non-alcoholic fatty liver disease by reducing hepatic lipid levels and mitigating lipid-induced oxidative injuries. Int. Immunopharmacol..

[41] Gao J., Chen S., Qiu Z., Fang L., Zhang L., Guo C., Chen T., Qiu L. (2018). Myricitrin ameliorates ethanol-induced steatosis in mouse AML12 liver cells by activating AMPK, and reducing oxidative stress and expression of inflammatory cytokines. Mol. Med. Rep..

[42] Li H.-Y., Gan R.-Y., Shang A., Mao Q.-Q., Sun Q.-C., Wu D.-T., Geng F., He X.-Q., Li H.-B. (2021). Plant-Based Foods and Their Bioactive Compounds on Fatty Liver Disease: Effects, Mechanisms, and Clinical Application. Oxid. Med. Cell Longev..

[43] Bagherniya M., Nobili V., Blesso C.N., Sahebkar A. (2018). Medicinal plants and bioactive natural compounds in the treatment of non-alcoholic fatty liver disease: A clinical review. Pharmacol. Res..

[44] Allaire M., Rautou P.E., Codogno P., Lotersztajn S. (2019). Autophagy in liver diseases: Time for translation?. J. Hepatol..

[45] Kwanten W.J., Martinet W., Michielsen P.P., Francque S.M. (2014). Role of autophagy in the pathophysiology of nonalcoholic fatty liver disease: A controversial issue. World J. Gastroenterol..

[46] Lee J.M., Wagner M., Xiao R., Kim K.H., Feng D., Lazar M.A., Moore D.D. (2014). Nutrient-sensing nuclear receptors coordinate autophagy. Nature.

[47] Kim S.H., Kim G., Han D.H., Lee M., Kim I., Kim B., Kim K.H., Song Y.M., Yoo J.E., Wang H.J. (2017). Ezetimibe ameliorates steatohepatitis via AMP activated protein kinase-TFEB-mediated activation of autophagy and NLRP3 inflammasome inhibition. Autophagy.

[48] Rusten T.E., Stenmark H. (2010). p62, an autophagy hero or culprit?. Nat. Cell Biol..

[49] Kang R., Zeh H.J., Lotze M.T., Tang D. (2011). The Beclin 1 network regulates autophagy and apoptosis. Cell Death Differ..

